# Differences in allergen-induced T cell activation between allergic asthma and rhinitis: Role of CD28, ICOS and CTLA-4

**DOI:** 10.1186/1465-9921-12-25

**Published:** 2011-02-28

**Authors:** Karine Botturi, Yannick Lacoeuille, Arnaud Cavaillès, Daniel Vervloet, Antoine Magnan

**Affiliations:** 1 INSERM UMR915, Nantes, F-44000 France; 2 Université de Nantes, Faculté de Médecine, l'institut du thorax, Nantes, F-44000 France; 3 CHU Nantes, l'institut du thorax, Service de pneumologie, Nantes, F-44000 France; 4 Service de Pneumo-allergologie, Hôpital Sainte Marguerite, Assistance Publique Hôpitaux de Marseille, Marseille, France

## Abstract

**Background:**

Th2 cell activation and T regulatory cell (Treg) deficiency are key features of allergy. This applies for asthma and rhinitis. However with a same atopic background, some patients will develop rhinitis and asthma, whereas others will display rhinitis only. Co-receptors are pivotal in determining the type of T cell activation, but their role in allergic asthma and rhinitis has not been explored. Our objective was to assess whether allergen-induced T cell activation differs from allergic rhinitis to allergic rhinitis with asthma, and explore the role of ICOS, CD28 and CTLA-4.

**Methods:**

T cell co-receptor and cytokine expressions were assessed by flow cytometry in PBMC from 18 house dust mite (HDM) allergic rhinitics (R), 18 HDM allergic rhinitics and asthmatics (AR), 13 non allergic asthmatics (A) and 20 controls, with or without anti-co-receptors antibodies.

**Results:**

In asthmatics (A+AR), a constitutive decrease of CTLA-4+ and of CD4+CD25+Foxp3+ cells was found, with an increase of IFN-γ+ cells. In allergic subjects (R + AR), allergen stimulation induced CD28 together with IL-4 and IL-13, and decreased the proportion of CTLA-4+, IL-10+ and CD4+CD25+Foxp3+ cells. Anti-ICOS and anti-CD28 antibodies blocked allergen-induced IL-4 and IL-13. IL-13 production also involved CTLA-4.

**Conclusions:**

T cell activation differs between allergic rhinitis and asthma. In asthma, a constitutive, co-receptor independent, Th1 activation and Treg deficiency is found. In allergic rhinitis, an allergen-induced Treg cell deficiency is seen, as well as an ICOS-, CD28- and CTLA-4-dependent Th2 activation. Allergic asthmatics display both characteristics.

## Background

Atopic diseases including allergic rhinitis and asthma are inflammatory conditions that have increased in prevalence over the past two decades [1]. The inflammatory response to common environmental allergens during allergy and asthma has been extensively studied in the past years, and has clearly determined the pivotal role of T cell activation, with a predominant Th2 cytokine production [2,3]. T regulatory (Treg) cells, characterized by the production of anti-inflammatory cytokines such as IL-10 and TGF-β [4,5] are considered as responsible for the normal tolerance against auto-antigens and external antigens such as allergens [6]. Accordingly, a deficiency in Treg counts and activation was found in autoimmune diseases and allergic conditions, notably during allergen exposure [7,8] and exacerbations of severe asthma [9]. However although this Th2/Treg imbalance applies both for allergic rhinitis and asthma, it is remarkable that despite a same atopic background and allergen exposure, some subjects will develop both rhinitis and asthma whereas other will display rhinitis only. We hypothesize since several years that T cell activation is different between both conditions and with others we previously described a Th1 activation in asthma that was absent in non asthmatic allergy in blood, induced sputum and broncho-alveolar lavages [10-12]. However, the role of allergen in the tuning of T cell activation in allergic rhinitics with and without asthma was not explored yet.

Allergen-induced T cell activation depends on signals delivered from antigen presenting cells (APCs) through the antigen-specific T cell receptor as well as additional co-stimulatory signals provided by engagement of so-called co-receptors on APCs and T cells [13]. Major T cell co-receptors are CD28, inducible costimulatory molecule (ICOS) and cytotoxic T lymphocyte antigen (CTLA)-4. They belong to the immunoglobulin gene superfamily and display various kinetics of expression. CD28 is a constitutive co-stimulatory receptor binding CD80 and CD86 on APCs, delivering important signals for T cell activation and survival. Ligation of CD28 promotes the production of IL-4 and IL-5 and provides resistance to apoptosis and long-term expansion of T-cells. As CD28, ICOS is a positive regulator of T cell activation which is up-regulated on activated T-cells. ICOS was initially shown to selectively induce high levels of IL-10 and IL-4, but is also able to stimulate both Th1 and Th2 cytokine production *in vivo *[14].

CTLA-4 is also a CD80/CD86-binding protein. It is up-regulated on activated T cells and delivers mainly an inhibitory signal, playing an important role in maintenance of peripheral tolerance [15]. Indeed, it was shown in murine Treg cells, that CTLA-4 controlled homeostasis and suppressive capacity of regulatory T cells [16].

Co-receptors thus represent important potential targets for therapeutic immunomodulation. Indeed the blockade of CD28 and CTLA-4 agonists are tested for their ability to prevent graft rejection [17], and in animal models, ICOS inhibition prevented allergic inflammation [18]. However, the actual role of co-receptors in the context of asthma and allergy in humans is still unexplored.

The objective of this study was therefore to compare the pattern of T cell activation between allergic rhinitics and asthmatics upon allergen stimulation and to assess the role of co-receptors CD28, ICOS and CTLA-4 in this process.

## Methods

### Study population

Four groups of patients were recruited: allergic rhinitics (R), allergic rhinitics and asthmatics (AR), non allergic asthmatics (A), and controls (C). All allergic patients were selected to display house dust mite (HDM) allergy. As rBetv1 birch pollen allergen was used as control antigen for *in vitro *stimulation of T cells, patients were selected to be not sensitized to birch pollen. The diagnosis of HDM allergy was determined by positive skin prick test to *Dermatophagoides pteronyssinus *extract (Stallergenes, France). Allergic rhinitis was defined by the presence of perennial nasal symptoms out of viral infection such as nasal obstruction, sneezing, rhinorrhea and nasal pruritus. The diagnosis of asthma was done on the basis of a history of dyspnea and wheezes with a reversible obstructive ventilatory defect or a positive methacholine challenge. The distinction between mild and moderate asthma was done according to GINA classification [19]. In patients, any inhaled corticosteroids and anti-histamines were discontinued 15 days before sampling. As controls, healthy non smoker individuals with normal lung function, negative methacholine challenge and negative skin prick test were included. In controls, absence of allergy was established by the negativity of 35 skin prick tests to common environmental aeroallergens, and absence of asthma was stated on negative methacholine challenge and induced sputum eosinophil count below 3% (see additional file 1: Skin testing, methacholine challenge and induced sputum procedures). The positive methacholine test was defined by a drop of at least 20% of FEV1(forced expiratory volume in 1 second) in response to 200 μg or less of metacholine. This project was approved by the local Ethic Committee and written informed consent was obtained from each patient.

### Isolation of PBMC

Peripheral blood mononuclear cells (PBMC) were isolated from peripheral venous blood by Ficoll-Hypaque plus (GE Healthcare, Uppsala, Sweden) density gradient centrifugation. Cells were then washed three times and resuspended in complete medium RPMI-1640 supplemented with 10% (v/v) foetal calf serum (FCS), 2 mM L-glutamine, 1 mM sodium pyruvate, 1 μM 2-mercapto-ethanol (Sigma Chemical, Saint-Louis, Missouri), 1000 U/ml Penicillin and Streptomycin. All culture reagents, except 2-mercapto-ethanol, were purchased from GIBCO^®^.

### Antigens

Recombinant (r) Betv 1 of birch pollen (*Betula verrucosa*) and purified (p) Derp 1 of house dust mite (*Dermatophagoides pteronyssinus*) were provided by Stallergènes (Antony, France). None of the allergens contained detectable amounts of LPS.

### Specific stimulation of T cells

Optimal dose of stimulatory pDerp1 and kinetics of T cell cytokine secretion and proliferation were determined in an independent pilot study on 5 house dust mite allergics and 5 healthy volunteers.

PBMC (5 × 10^5^) were cultured in 96 wells plates (Falcon) in 100 μl medium containing 1 μg/ml pDerp1 at 37°C in 5%CO_2 _and cells were harvested after 8 days culture. 50 μl of fresh complete medium was added every 2 days in each well. rBetv1 was used as control antigen at a concentration of 1 μg/ml.

### Surface staining

After 8 days of culture with pDerp 1, PBMC (5 × 10^5^) were harvested and stained with anti-CD4-PE-Cy5, anti-CD25-FITC, (Beckman Coulter, Marseille, France); anti-CD3-FITC (Dako, Trappes, France), anti-CD3-PE-Cy5 (Immunotools, Friesoythe, Germany), anti-CD28-FITC, anti-ICOS-PE, or anti-CTLA-4-PE-Cy5 (BD Pharmingen, le Pont de Claix, France) mAbs at recommended concentrations. To detect Foxp3 intracellular transcription factor, T cells were then fixed, permeabilized, and stained with anti-Foxp3-PE mAb (eBiosciences, San Diego, California). The Treg population was identified as CD4+CD25^Hi^+Fox p 3+ cells.

Fluorescence was detected with a 15 mW argon ion laser on a three colors FACSCan^® ^(Becton Dickinson, Franklin Lakes, NJ, USA). Standard acquisition and analysis software were obtained through Cellquest^® ^Software (Becton Dickinson).

### Intracellular T cell cytokine staining

PBMC (5 × 10^5^) were cultured for 8 days with pDerp 1. PMA (Sigma Chemical, Saint-Louis, Missouri, 50 ng/ml), Ionomycin (Euromedex, 2 μg/ml) and Monensin (Sigma Chemical, 2 μM) were added during the last 6 hours of culture. These culture conditions allow the detection of cytokines already engaged in a synthesis process in vivo [20]. Cells were harvested and stained with CD3-PE-Cy5 (Immunotools, Friesoythe, Germany). Cells were then fixed, permeabilized, and stained with antibodies to detect intracellular cytokines (anti-IFNγ-FITC, anti-IL-4-FITC, BD Pharmingen, le Pont de Claix, France; anti-IL13-PE, anti-IL-10-PE, R&D system, Lille, France). IL-4+ and IL-13+ cells were considered as Th2 cells, IFN-γ + cells as Th1 cells. IL-10+ cells were considered as belonging to Treg cell population.

### Co-receptor study

To determine the role of co-receptors in T cell activation, PBMC cultures were performed with or without anti-CTLA-4 (clone 14D3, 12 μg/ml), anti-ICOS (clone ISA-3, 12 μg/ml) or anti-CD28 (clone CD28.6, 3 μg/ml) monoclonal antibodies (mAb). These mAb were purchased from eBioscience.

### Statistical Analysis

Analysis was performed using the Statview^® ^Software. Normal distributions of the variables were checked with a Kolmogorov-Smirnof's test. Average percentages of positive cells and cytokine concentrations were then compared between groups (controls, non allergic asthmatics, allergic rhinitics and allergic asthmatics) using the analysis of variance (ANOVA). When the ANOVA showed statistical difference between groups, a multiple linear regression analysis was done to identify if allergy, asthma, or both could explain the variable studied. Between-groups comparisons were performed using a Student's t-test. A paired t test was used to compare differences between paired groups. A p value < 0.05 was considered as statistically significant for all statistical tests. Results are expressed as mean ± standard error (SE).

## Results

### Study population

Sixty-nine subjects (33 males, 36 females, mean age 37.20 ± 1.90) were included. Blood samples from 20 healthy individuals with no history of allergy or asthma, 18 allergic asthmatics (AR), 18 allergic rhinitics (R), and 13 non allergic asthmatics (A) were collected. Characteristics of the patients are shown in table 1.

**Table 1:** Characteristics of the patients

	Controls (n = 20)	R (n = 18)	AR (n = 18)	A (n = 13)
**Clinical Data**				
Gender, (M/F)	5/15	12/6	10/8	6/7
Age*	32.38 ± 4.49	33.68 ± 3.23	40.56 ± 3.59	50.61 ± 4.36
Body Mass Index	24 ± 0.5	22.5 ± 1.5	25 ± 4	24.5 ± 2
mild/moderate asthma	-	-	9/9	6/7
**Lung function**				
FEV_1_, (% of theoretical value*)	100 ± 2.26	97.4 ± 4.30	89.5 ± 3.91	83.8 ± 6.64
Sputum eosinophils (%)*	0.5 ± 0.31	0.75 ± 0.75	14.78 ± 5.98**	29 ± 9.99**

FEV_1 _= Forced expiratory volume in 1 second

* Values are mean ± standard error (SE),

** = p < 0.01. R = allergic rhinitis; A R = allergic asthma and rhinitis; A = non allergic asthmatics.

None of the subjects was a smoker. Patients interrupted their local or systemic steroids or antihistamines 15 days before sampling. Asthmatics were mild asthmatics for one half and moderate asthmatics for the other half. All allergic patients displayed symptoms compatible with allergic rhinitis. All non allergic asthmatics also complained from nasal symptoms. Healthy volunteers did not report any symptom.

Sputum eosinophil counts were significantly higher in asthmatics than in control subjects or allergic rhinitis, with no significant difference between allergic and non allergic asthmatics. None of the subjects was sensitized to birch. The age difference between the A+R group and other groups (A, R and C) was not significant statistically.

### T cell activation and co-receptor expression before specific stimulation

Treg cells proportion, Th1 and Th2 cytokines production and co-receptors expression (CTLA-4, ICOS, CD28) in each group were first assessed by flow cytometry, prior to any specific stimulation.

**Figure 1:**
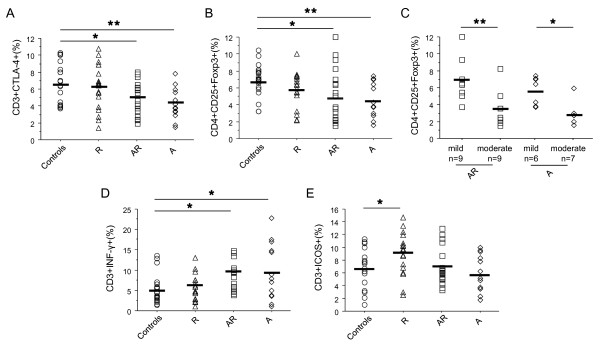
**T cell activation and co-receptor expression before specific stimulation**. CTLA-4 expression (A), Treg cells (CD4+CD25+^Hi^Foxp3+, B), IFN-γ producing T cells (D) and ICOS expression (E) were assessed by flow cytometry in PBMC from HDM allergic rhinitics (R) (triangle, n = 18), allergic asthmatics and rhinitics (AR) (square, n = 18), non allergic asthmatics (A) (lozenge, n = 13), and controls (circle, n = 20). Treg cells were also evaluated in non allergic asthma and allergic asthma between mild and moderate asthmatics (C). Results are expressed as percentage of total T cell and compared versus controls. _ : mean of each group. * = p < 0.05; ** = p < 0.01

In non-stimulated conditions, CTLA-4+ T cells were decreased in asthmatics (p < 0.05 vs controls, figure 1A), whatever their allergic status. In keeping with this result, a reduced Treg population (p < 0.025, figure 1B) was found in these patients. Relevantly, Treg cell proportions were higher in mild asthmatics than in moderate counterparts (p < 0.012, figure 1C). IFN-γ + cells were increased (p < 0.022 vs controls, figure 1D) in asthmatics. No significant difference in Th2 cytokines or IL-10 production was found (table 2) between groups.

**Table 2:** Baseline T-cell co-receptor and cytokine expression

	Controls (n = 20)	R (n = 18)	AR (n = 18)	A (n = 13)
CD3+IL-4+ (%)	2.38 ± 0.26	2.88 ± 0.29	2.35 ± 0.22	3.25 ± 0.60
CD3+IL-13+ (%)	3.02 ± 0.31	4.17 ± 0.48	3.54 ± 0.34	4.18 ± 0.54
CD3+IL-10+ (%)	5.01 ± 0.47	4.06 ± 0.33	4.38 ± 0.37	4.37 ± 0.46
CD3+CD28+ (%)	87.75 ± 2.50	89.04 ± 1.71	89.50 ± 1.31	84.85 ± 3.45

PBMC from each patient were cultured in complete medium during 8 days. ICOS, CD28, IL-4, IL-13, and IL-10 expression by T-cells were assessed by flow cytometry. Results are expressed as mean of total T-cells ± SE and compared versus controls. * = p < 0.05, ** = p < 0,01. R = allergic rhinitis; A R = allergic asthma and rhinitis; A = non allergic asthmatics.

ICOS expression was higher in R compared to controls (p = 0.029, figure 1E), but similar in AR and controls. No significant variation was found at the level of CD28 expression between groups (table 2).

The multiple linear regression analysis showed that asthma (A + AR) was associated to lower ICOS and CTLA-4 expression and Treg cell proportions, but to higher IFN-γ+ T cells (table 3). By contrast, allergic rhinitis (with or without asthma) was positively linked to ICOS expression.

**Table 3:** Multiple linear regression analysis between asthma, allergy and allergy after specific stimulation

	Asthma (A+AR)	Allergy (R+AR)	Allergy + specific stimulation (R+AR+Derp1 stimulation)
CD3+ICOS+ (%)	**-1.502 **± **0.75 (p = 0.0485)**	**1.675 ± 0.75 (p = 0.0292)**	**2.929 ± 0.81 (p = 0.0006)**
CD3+CTLA-4+ (%)	**-1.649 ± 0.61 (p = 0.0087)**	0.508 ± 0.61 (p = 0.4088)	**-1.406 ± 0.60 (p = 0.0223)**
CD3+IL-4+ (%)	0.188 ± 0.34 (p = 0.585)	-0.066 ± 0.34 (p = 0.848)	**1.12 ± 0.37 (p = 0.034)**
CD3+IL-13+ (%)	0.287 ± 0.43 (p = 0.508)	0.429 ± 0.43 (p = 0.325)	**2.209 ± 0.43 (p < 0.0001)**
CD3+IFN-γ+ (%)	**3.3643 ± 1.63 (p = 0.0283)**	1.44 ± 1.62 (p = 0.378)	0.884 ± 1.42 (p = 0.535)
CD4+CD25^Hi^+Foxp3+ (%)	**-1.647 ± 0.54 (p = 0.0033)**	-0.371 ± 0.54 (p = 0.494)	-0.774 ± 0.52 (p = 0.142)
CD3+IL-10+ (%)	-0.146 ± 0.42 (p = 0.729)	-0.549 ± 0.42 (p = 0.196)	**-1.896 ± 0.44 (p < 0.0001)**

Values are expressed as coefficient of regression ± SE

### T cell activation and co-receptor expression after specific stimulation by allergens

PBMCs were cultured in the presence or not of pDerp1 during 8 days. T cell activation and co-receptors expression were then studied by flow cytometry.

**Figure 2:**
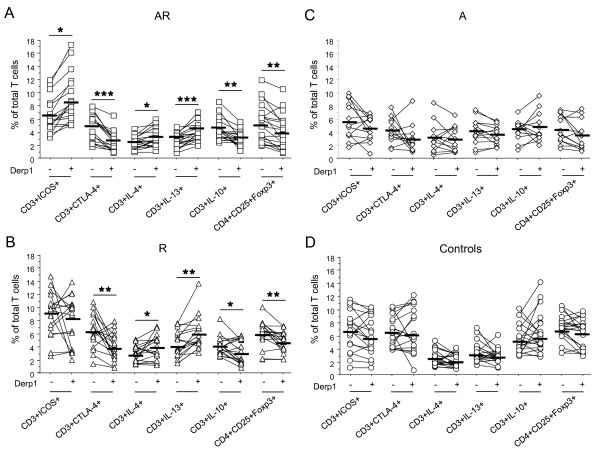
**T cell activation and co-receptor expression after specific stimulation**. ICOS, CTLA-4 expression, IL-4, IL-13, IL-10 producing T cells and Treg cells (CD4+CD25+^Hi^Foxp3+) were assessed by flow cytometry in PBMC from HDM allergic asthmatics and rhinitics (AR) (A, n = 18), HDM allergic rhinitics (R) (B, n = 18), non allergic asthmatics (A) (C, n = 13), and controls (D, n = 20) stimulated or not with Derp1 allergen (1 μg/ml) during 8 days. Results are expressed as percentage of total T cells. _ : mean of each group. * = p < 0.05; ** = p < 0.01

In AR, Der p 1 up-regulated CD28 (89.78 ± 1.33 vs 91.01 ± 1.48; p = 0.0016) and ICOS expression, and decreased CTLA-4 (figure 2A). Furthermore, Derp1 stimulation induced an increase in IL-4+ and IL-13+ cells (figure 2A), without significant variation in IFN-γ+ cells (not shown). This increase in Th2 cells was associated to a decrease in IL-10+ cells and Treg cells (figure 2A).

In R, Derp1 also increased CD28 (89.03 ± 1.71 vs 91.00 ± 1.48; p = 0.0025) but not ICOS expression (figure 2B). It decreased CTLA-4+ cell proportions. Allergen stimulation induced an increase in Th2 cells without variation of IFN-γ + cells (not shown), and a decrease in IL10+ and Treg cells (figure 2B).

Therefore at the exception of ICOS, that was already increased at baseline in R and thus could not increase upon stimulation, the profile of T cell activation and co-receptor expression induced by Derp1 was similar in AR and R subjects.

After specific stimulation (figure 2C-D), T cells from asthmatic and non asthmatic allergics displayed higher expression of ICOS (p < 0.02) and lower expression of CTLA-4 compared to controls (p < 0.007). In addition Th2 cell proportions were higher in allergics whereas Treg cells were decreased (IL-4, p < 0.0022; IL-13, p < 0.0001; Treg, p < 0.008). CD28+ cell percentages were not different between groups after allergen-specific stimulation (not shown). In non allergic subjects (figure 2C-D) no significant variation was found in any of the parameters studied

The multiple linear regression analysis showed that after Derp 1 specific stimulation, allergy (R + AR) correlated positively with percentages of ICOS, IL-13 and IL-4-expressing T cells and negatively with CTLA-4 and IL-10-expressing T cells (table 3).

No variation was found in any subject for any co-receptor or cytokine expression after stimulation with irrelevant rBetv1 (not shown).

### Role of co-receptor engagement

In order to study the respective role of CD28, ICOS and CTLA-4 in T cell activation patterns in the context of allergen presentation, PBMC were stimulated with Derp1 in the absence or presence of anti-ICOS, anti-CTLA-4 or anti-CD28 mAb.

**Figure 3:**
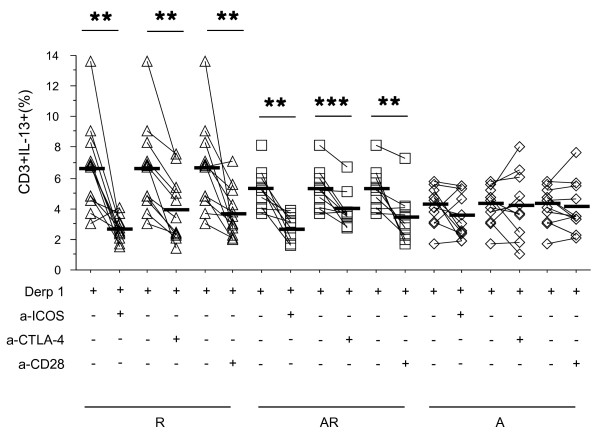
**Effect of anti-co-receptors antibodies on IL-13 production by T cells**. PBMC from allergic rhinitics (R) (triangle, n = 12), allergic rhinitic and asthmatics (AR) (square, n = 10) and non allergic asthmatics (A) (lozenge, n = 11) were stimulated with Derp 1 and cultured in the presence or absence of anti-ICOS, anti-CTLA-4 or anti-CD28 antibodies. IL-13 expressing T cells were then compared in each group versus baseline. Results are expressed as percentage of total T cells. Black line : mean of each group. * = p < 0.05; ** = p < 0.01; *** = p < 0.001

In allergics, whatever the asthmatic status (R + AR), anti-ICOS and anti-CD28 mAb specifically decreased IL-4+ and IL-13+ cells (figure 3A and table 4), but had no influence on IFN-γ+ cells (table 4). Anti-CTLA-4 mAb had no effect on IL-4+ cells, but unexpectedly decreased IL-13+ cell proportions (table 4).

**Table 4:** Effect of anti-co-receptors antibodies on Treg cells, IL-10 and IFN-γ production

	R (n = 12)				AR (n = 10)				A (n = 11)			
	**Derp1**	**Derp1+ anti-ICOS**	**Derp1+ anti-CTLA4**	**Derp1+ anti-CD28**	**Derp1**	**Derp1+ anti-ICOS**	**Derp1+ anti-CTLA4**	**Derp1+ anti-CD28**	**Derp1**	**Derp1+ anti-ICOS**	**Derp1+ anti-CTLA4**	**Derp1+ anti-CD28**

CD3+IL-10+(%)	3.82 ± 0.49	3.79 ± 0.30	4.66 ± 1.05	3.31 ± 0.32	3.80 ± 0.41	3.54 ± 0.46	3.96 ± 0.50	3.60 ± 0.52	5.03 ± 0.68	4.42 ± 0.56	4.42 ± 0.64	4.37 ± 0.64
CD4+CD25^Hi^+ Foxp3+ (%)	4.25 ± 0.45	4.49 ± 0.65	4.24 ± 0.57	3.57 ± 0.65	3.61 ± 0.70	4.21 ± 1.13	3.87 ± 0.83	2.78 ± 0.83	2.65 ± 0.47	2.47 ± 0.38	2.55 ± 0.40	1.75 ± 0.29
CD3+IFN-γ+ (%)	7.48 ± 1.15	6.13 ± 0.75	7.24 ± 1.11	7.63 ± 0.98	12.26 ± 1.35	11.86 ± 1.01	13.99 ± 1.84	13.64 ± 1.36	11.90 ± 1.89	10.43 ± 1.78	12.35 ± 1.74	12.32 ± 1.98
CD3+IL-4+ (%)	4.22 ± 0.55	2.01 ± 0.26*	3.67 ± 0.94	2.33 ± 0.40*	3.29 ± 0.49	2.21 ± 0.15***	2.85 ± 0.34	2.41 ± 0.22**	3.53 ± 0.50	2.56 ± 0.54	2.92 ± 0.51	3.11 ± 0.95

PBMC from allergic non asthmatics (R, n = 12), allergic asthmatics (AR, n = 10) and non allergic asthmatics (A, n = 11), were stimulated with Derp 1 and cultured in the presence or absence of anti-ICOS, anti-CTLA4 or anti-CD28 antibodies. Treg cells, IL-10 and IFN-γ production by T-cells were assessed by flow cytometry. Results are expressed as mean of total T-cells ± SE and compared versus absence of anti-co-receptors antibodies conditions. R = allergic rhinitis; A R = allergic asthma and rhinitis. * = p < 0.05, ** = p < 0,01, *** = p < 0,001.

In non allergic subjects (A + controls), anti-co-receptor antibodies did not affect Th1 or Th2 cytokine production (figure 3 and table 4).

## Discussion

The results of our *ex vivo *study strongly suggest a contrasted picture of T cell activation in allergic rhinitis and asthma, with distinct patterns of Th1, Th2 and Treg profiles and expression of ICOS, CD28 and CTLA-4 co-receptors.

Indeed, we showed that in asthma, IFN-γ production was constitutive, did not increase upon allergen stimulation, and was not blocked by any of the anti-co-receptor antibodies. Similarly, the constitutive defect of Treg and CTLA-4 expression seen in asthmatics and not enhanced in non allergic asthmatics after allergen stimulation was not modified after co-receptors blockade. The Th1/Treg imbalance in asthma is therefore constitutive and independent of allergen presentation.

The constitutive Th1 activation in asthma was demonstrated before [10,12,21]. It could result from the intrinsic defect in the CTLA-4+ and Treg populations as CTLA-4, known to be involved in tolerance induction [22], could prevent the asthmatic inflammation by inducing T cells to differentiate in T regulatory cells. Recently, we have showed during *in vivo *studies a lower proportion of Treg cells in blood from severe refractory asthmatics compared to controls, which was even deeper during exacerbations, both in blood and induced sputum [9]. Herein we show that this lower proportion of Treg is present in milder stages of asthma. Relevantly, Treg cells were higher in mild than in moderate asthma whatever the allergic status. This results are concordant with the primary Treg cell deficiency suggested in asthma and allergy [23]. That the Th1/Treg imbalance is similar in allergic and non allergic asthma suggests that it is a characteristic of asthma independent of allergy, possibly triggered by infectious agents or non specific substances such as pollutants but it must be precised that asthmatics included in the present study were controlled and did not experienced any recent exacerbation. Another hypothesis would be that the Th1/Treg imbalance in asthma is really intrinsic and independent of any external aggression.

In allergic groups, we demonstrated a Th2/Treg imbalance inducible upon allergen stimulation. That Th2 activation was not seen in non allergic patients and could be broken by CD28 and ICOS blockade indicates that it is really the cognate allergen presentation by antigen presenting cells that was responsible for it. IL-13 secretion was suppressed also by blocking CTLA-4, indicating that in peripheral cells (1) Th2 activation cannot be considered globally, Th2 cytokines being regulated distinctly, and (2) CTLA-4 being not only involved in tolerance but also in inflammation. This result is concordant with Lordan and al., who showed that allergen-induced production of IL-5 and IL-13 by PBMC from allergic asthmatics could be inhibited by blocking CTLA-4 receptor with CTLA-4-Ig [24]. Regarding the allergen-induced Treg defect in allergics, other co-receptors than these tested are likely involved, among which PD1 is a candidate [25]. Indeed, Meiler et al. recently demonstrated in PBMC from allergic patients that the suppressive effect of IL-10 secreting T cells was partially inhibited by blocking CTLA-4 or PD-1 co-receptors, whereas blocking both receptors simultaneously had an additive effect [26].

The association of allergy with ICOS over-expression before any allergen stimulation suggests a non specific priming of T cells towards the Th2 pathway in allergic subjects. Indeed ICOS was clearly related to Th2 activation, as shown by anti-ICOS stimulation results. Numerous studies using animal models of airways inflammation have showed that ICOS-mediated signalling was essential for induction of Th2 cytokines [27,28]. Indeed inhibition of ICOS suppresses allergic lung inflammation and Th2 cytokines production in mice models [29]. However in other models ICOS engagement induces tolerance and inhibits the allergic inflammation. These distinct actions of ICOS seem related to the density of ICOS molecules per cell, with inflammation being related to a high density of co-receptors and tolerance induction to a lower number of ICOS molecules per cell [30].

That in R ICOS expression does not increase after allergen stimulation by contrast with the AR group could result from a maximal expression of ICOS in R whereas it is still inducible in AR. Indeed the basal level of ICOS expression is lower in the latter group than in the former. This relative defect in ICOS expression in AR patients could result from the constitutive Th1/Treg imbalance of asthmatics that by a Th1-driven "anti-Th2" effect would decrease ICOS expression.

Under allergen stimulation, CD28 expression increased significantly in R and AR, and blockade of CD28 decreased the Th2 cytokine production, indicating the involvement of CD28 in Th2 cell activation in allergy. It is noteworthy that although significant statistically, the proportion of CD28+ cells could not increase in high proportion, as most T cells constitutively expressed CD28 in all groups. CD28 is a crucial co-receptor for inducing T cell cytokine production [31], and was showed to be involved both in Th1 and Th2 activation. CD28 blockade is proposed as an immunosuppressive strategy to prevent graft rejection, and is experimented in various inflammatory diseases. However the practical use of CD28 blockade was refrained by the agonist action of some anti-CD28 antibodies encountered in clinical trials [32].

Our study provides new insights into the hypothesis of Treg cell deficiency as a paradigm for allergic diseases, by showing a constitutive Treg cell deficit in asthma whatever the allergic status and an inducible Treg deficit in allergy, whatever the presence of asthma. As a consequence, the Treg cell deficiency is the highest in asthmatic allergics after allergen stimulation. This distinction between allergy and asthma contradicts our previous hypothesis of a gradient of Treg cell deficiency from allergy to asthma [23], and better suggests that the abnormalities seen in both diseases could be juxtaposed and independent, as showed by the multiple linear regression analysis.

Recently an *in vivo *study showed no difference in the number of Treg cells between asthmatics and controls, whereas FOXP3 protein expression within Treg cells was significantly decreased in asthmatic patients [33]. Our study was performed in blood *ex vivo *and therefore might not fully reflect the *in vivo *and in situ reality. However many studies showed that blood compartment was relevant to the in situ inflammation as far as T cells and allergy were concerned [21], and the mechanistic studies proposed here cannot be assessed in situ in humans. They can be performed *in vivo *in animals, but the relevance to real asthma would also be uncertain.

In conclusion, allergy is associated to a constitutive ICOS over-expression and inducible CTLA-4 under-expression with Th2/Treg imbalance, when a constitutive CTLA-4 under-expression and Th1/Treg disequilibrium appears as a hallmark of asthma. Both profiles are mixed in allergic asthma, and one can argue that asthma would occur in allergic subjects only if the unknown conditions leading to the constitutive Th1 activation are present. Still missing in the puzzle is the stimulus inducing the Th2 activation present in non allergic asthma [3]. Lastly, our results demonstrate that although targeting one type of T cell activation only would be a pitfall in allergic asthma, there is a rationale to develop strategies based on targeting co-receptors in allergy.

## Conclusion

In conclusion, our work adds significant insights into the immune mechanism involved in allergy and asthma and states the rationale for new diagnosis and/or therapeutic strategies in these pathologies.

## Competing interests

The authors declare that they have no competing interests.

## Authors' contributions

All the authors have contributed significantly to the research and preparation of the manuscript, and they approve its submission.

## Supplementary Material

Additional file 1 **Skin testing, methacholine challenge and induced sputum procedures**. Click here for file

## Aknowledgements

We kindly acknowledge Stallergènes for providing the Der p 1 purified protein and Betv1 recombinant protein.
