# Supplementary material for: Differences in allergen-induced T cell activation between allergic asthma and rhinitis: Role of CD28, ICOS and CTLA-4
Source: Respir Res. 2011 Feb 28;12(1):25. doi: 10.1186/1465-9921-12-25 (PMC3051906; doi:10.1186/1465-9921-12-25)
Supplement: Additional file 1 — Skin testing, methacholine challenge and induced sputum procedures. [file 1465-9921-12-25-S1.DOC]

**Skin testing, methacholine challenge and induced sputum procedures**

# **Skin testing**

The diagnosis of atopy was allowed if at least one skin prick test to a common environmental aeroallergen from a standard battery of 35 extracts (Laboratoire des Stallergenes, Paris, France) was positive. The skin test battery included the following allergens: house dust (100 IR, i.e., index of reactivity that is a concentration which induced a 7-mm wheal in a group of sensitized individuals), *Dermatophagoides* *pteronyssinus* and *farinae* (100 IR), feather (1/10 wt/vol), cat and dog extracts (100 IR), German cockroach (1/10 wt/vol), *Alternaria*, *Aspergillus*, *Cladosporium*, and *Penicillium* (1/10, wt/vol), mixed grass pollen (poa, fescue, timothy, rye, orchard) (100 IR), Bermuda grass (1/20 wt/vol), *Parietaria* (1/20 wt/vol), alders (100 IR), birch tree (100 IR), hornbeam, hazel, olive tree, ash, privet, oak, mimosa, poplar, false acacia, lime tree, mulberry, nettle tree (1/20 wt/vol), mixed weeds (100 IR) including ragweed, mugwort, amaranth, goosefoot, sorrel, and plantain. Prick tests were considered positive if the wheal diameter 20 min after allergen injection reached at least half the wheal diameter induced by codeine phosphate and more than the wheal induced by saline 9%. In any case, a positive (codeine phosphate 9%) and a negative test were performed.

**Methacholine challenge**

Methacholine challenge tests were performed when spirometric data were normal. Cumulative doses of methacholine were administered through a dosimeter (ME-FAR dosimeter; Elletromedically, Brescia, Italy); specific airway resistance (SRaw) and FEV1 measurements were made after each dose in an 830-L constant body plethysmograph (model Master Lab Jaeger, Wurzburg, Germany). Bronchial hyperreactivity was defined by a 100% increase of SRaw at 200 mg of methacholine or less. The dose of methacholine inducing a decrease of 20% of the FEV1 (PD20) was determined for each positive test.

**Induced sputum procedures**

Sputum was induced with hypertonic saline (4.5%) aerosol during three successive 7 min periods. Immediately, cellular plugs were separated from saliva, and incubated with 0.1% dithiothreitol. Cells were filtered through a 40 micron cell strainer, washed, and resuspended in RPMI (Gibco) at a concentration of 1.10^6^ cells/ml. Total nucleated cell counts and viability were assessed after trypan blue exclusion. Samples were validated by a viability > 50 % and a salivary cell contamination < 20%, according to European Respiratory Society guidelines [1].

References in additional file:

1. Djukanovic R, Sterk PJ, Fahy JV, Hargreave FE: **Standardised methodology of sputum induction and processing**. *Eur Respir J Suppl* 2002, **37:**1s-2s.
